# Effect of ceritinib (LDK378) on enhancement of chemotherapeutic agents in ABCB1 and ABCG2 overexpressing cells *in vitro* and *in vivo*

**DOI:** 10.18632/oncotarget.5989

**Published:** 2015-11-09

**Authors:** Jing Hu, Xu Zhang, Fang Wang, Xiaokun Wang, Ke Yang, Meng Xu, Kenneth K.W. To, Qingshan Li, Liwu Fu

**Affiliations:** ^1^ Department of Hematology, Guangzhou First People's Hospital, Guangzhou Medical University, Guangzhou, China; ^2^ Collaborative Innovation Center for Cancer Medicine, State Key Laboratory of Oncology in South China, Guangdong Esophageal Cancer Institute, Cancer Center, Sun Yat-Sen University, Guangzhou, China; ^3^ School of Pharmacy, Faculty of Medicine, the Chinese University of Hong Kong, Hong Kong SAR, China

**Keywords:** ceritinib, multidrug resistance, ATP-binding cassette transporters, ABCB1, ABCG2

## Abstract

Multidrug resistance (MDR) is the leading cause of treatment failure in cancer chemotherapy. The overexpression of ATP-binding cassette (ABC) transporters, particularly ABCB1, ABCC1 and ABCG2, play a key role in mediating MDR by pumping anticancer drugs out from cancer cells. Ceritinib (LDK378) is a second-generation tyrosine kinase inhibitor of anaplastic lymphoma kinase (ALK) currently in phase III clinical trial for the treatment of non-small cell lung cancer. Here, we found that ceritinib remarkably enhanced the efficacy of chemotherapeutic drugs in ABCB1 or ABCG2 over-expressing cancer cells *in vitro* and *in vivo*. Ceritinib significantly increased the intracellular accumulation of chemotherapeutic agents such as doxorubicin (DOX) by inhibiting ABCB1 or ABCG2-mediated drug efflux in the transporters-overexpressing cells. Mechanistically, ceritinib is likely a competitive inhibitor of ABCB1 and ABCG2 because it competed with [^125^I]-iodoarylazidoprazosin for photo affinity labeling of the transporters. On the other hand, at the transporters-inhibiting concentrations, ceritinib did not alter the expression level of ABCB1 and ABCG2, and phosphorylation status of AKT and ERK1/2. Thus the findings advocate further clinical investigation of combination chemotherapy of ceritinib and other conventional chemotherapeutic drugs in chemo-refractory cancer patients.

## INTRODUCTION

Chemotherapy is the main treatment for many malignant tumors. Unfortunately, cancer cells develop multidrug resistance (MDR) to a variety of structurally and functionally unrelated anticancer drugs, thus hindering the otherwise successfully chemotherapy [1]. The most common mechanism of MDR is the overexpression of ATP binding cassette (ABC) transporters, which actively pump numerous chemotherapeutic drugs out from cancer cells, thereby attenuating the efficacy of chemotherapeutic agents. Up to now, 49 different ABC transporters have been identified in the human genome, which are divided into seven subfamilies (ABCA-ABCG) on the basis of amino acid sequence similarities and phylogeny [2]. ABC transporter can transport a wide range of chemotherapeutic drugs including the anthracyclines, vinca alkaloids, taxanes, and epipodophyllotoxins [3], and it is also a transporter that effluxes molecularly targeted drugs such as tyrosine kinase inhibitors (TKIs). Among them, the ABC transporter subfamily B member1 (*ABCB1*/MDR1/P-glycoprotein, P-gp), subfamily C member 1 (*ABCC1*/MRP1) and subfamily G member 2(*ABCG2*/BCRP) are considered to be the most important transporters to confer MDR to tumor cells. ABCB1 (MDR1/P-gp) was the first eukaryotic ABC transporter identified that confers MDR in cancer cells [4]. ABCG2 (BCRP/MXR) is the first half transporter in the ABC transporter family, it has been found in mitoxantrone (MX) selected human colon carcinoma cell line S1-M1-80, so giving ABCG2 the name of mitoxantrone resistant protein (MXR) [5]. ABCG2 can actively efflux a wide variety of chemotherapeutic drugs including organic anion conjugates, nucleoside analogs, organic dyes, TKIs, anthracyclines, camptothecin-derived indolocarbazole topoisomerase I inhibitors, methotrexate and flavopiridols [6]. Interestingly, ABCG2 was also found to be a determinant of side population (SP) cells that are highly enriched in cancer stem cells [7]. Thus, targeting ABCG2 in the tumor stem cells represents a promising and novel strategy to eradicate the entire cancer cell population. ABCC1 is a 190-kDa protein that was first discovered in DOX resistant HL60/Adr and H69AR cell lines [8, 9]. Its substrates include vinblastine (VLB), vincristine (VCR) and so on. Therefore, inhibition of ABC transporters may represent a promising strategy to circumvent MDR by increasing intracellular accumulation of chemotherapeutic drugs. Enormous effort has been devoted to the development of ABC transporter inhibitors. To date, three generations of MDR inhibitors have been developed, some of which are currently under clinical trials to evaluate their usefulness in circumventing anticancer drug resistance.

Tyrosine kinase inhibitors (TKIs) are a new class of molecular targeted chemotherapeutic drugs that selectively kill cancer cells by blocking key oncogenic pathways. Importantly, the recent discovery of potent and specific inhibition of several ABC transporters by TKIs has advocated their use as chemosensitizers to circumvent MDR. Ceritinib (LDK378/Zykadia) is a clinically approved second generation TKI specifically targeting the anaplastic lymphoma kinase (ALK). In preclinical setting, ceritinib was found to be more potent than the first generation ALK inhibitor crizotinib [10, 11]. It is indicated for the treatment of patients with ALK-positive and metastatic lung adenocarcinoma with disease progression, or who are intolerant to crizotinib. In this study, we investigated the effect of ceritinib on the circumvention of MDR *in vitro* and *in vivo*.

## RESULTS

### Cytotoxicity effect of ceritinib alone on MDR cells and their parental sensitive cells

**Figure 1:**
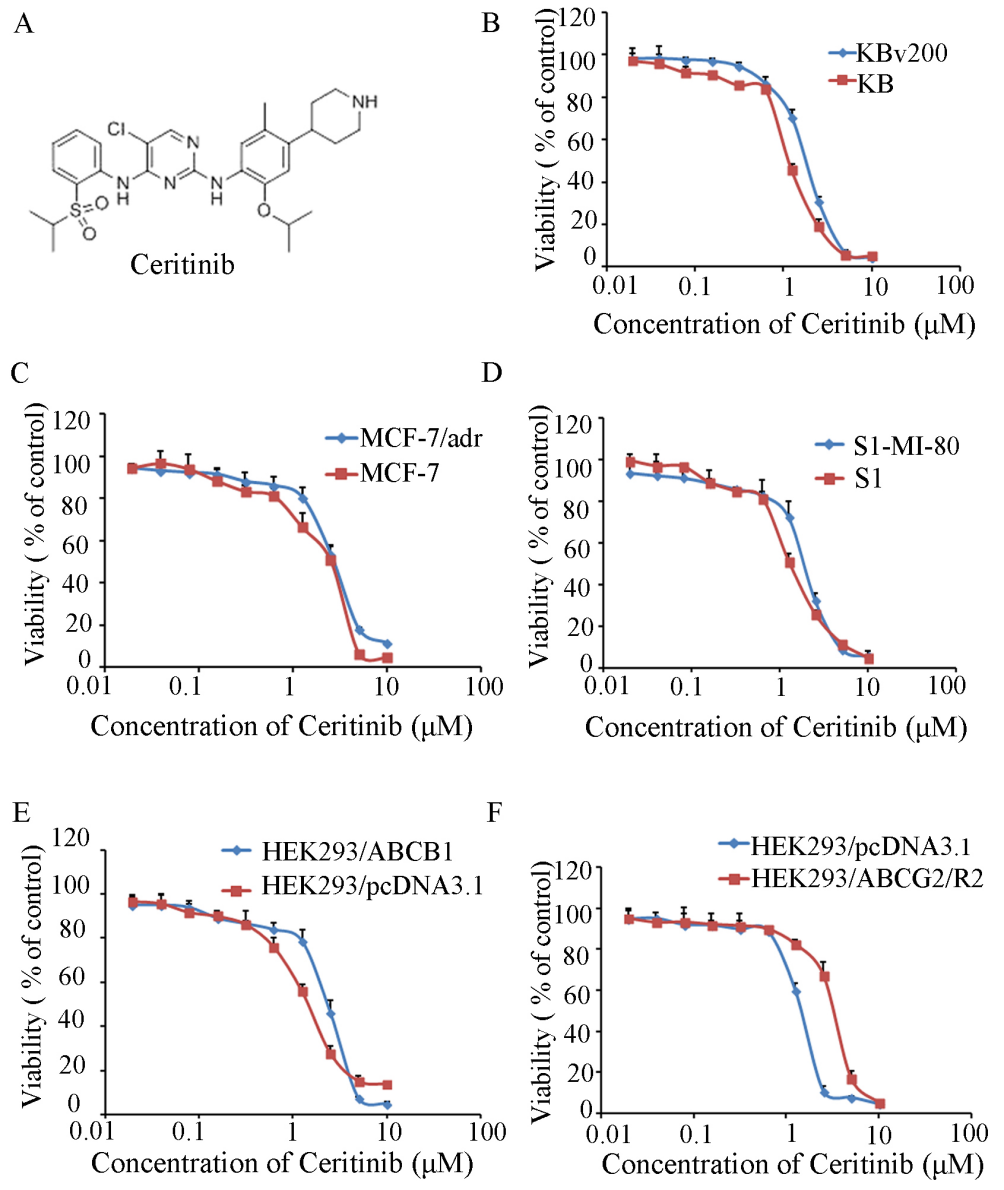
The structure of Ceritinib and cytotoxicity of Ceritinib **A.** the structure of Ceritinib; **B.** MTT cytotoxicity assay was assessed ABCB1 and ABCG2 over-expressing cells and their parental sensitive cells: ABCB1-negative KB and ABCB1-overexpressing KBv200 cells; **C.** ABCB1-negative MCF-7 and ABCB1-overexpressing MCF-7/adr cells; **D.** ABCG2-negative S1 and ABCG2-overexpressing S1-M1-80 cells; **E.** ABCB1-negative HEK293/pcDNA3.1 and ABCB1-overexpressing HEK293/ABCB1 cells; **F.** ABCG2- negative HEK293/pcDNA3.1 and ABCG2-overexpressing HEK293/ABCG2-R2 cells. All cells were treated with a range of different concentrations of ceritinib for 72 hours. Results from three independent experiments are presented as the mean ± SD.

The cytotoxicity of ceritinib in different cells was determined by the MTT assay. The IC_50_ values were 1.10 ± 0.31, 1.69 ± 0.41, 2.15 ± 0.33, 2.73 ± 0.46, 1.34 ± 0.35, 1.69 ± 0.39, 1.50 ± 0.37, 1.86 ± 0.34, 2.84 ± 0.56 for KB, KBv200, MCF-7, MCF-7/adr, S1, S1-M1-80, HEK293/pcDNA3.1, HEK293/ABCB1 and HEK293/ABCG2-R2, cells respectively. Based on the cytotoxicity curves, more than 85% of the cells survived at the concentration of 0.5 μM ceritinib (Figure 1B–1F). Therefore, 0.5 μM of ceritinib was chosen as a maximum concentration for combination treatment with ABCB1 and ABCG2 substrate anticancer drugs.

### Ceritinib potentiated the cytotoxicity of conventional chemotherapeutic agents in ABCB1 and ABCG2-overexpressing cells

The IC_50_ values of the anticancer drugs in sensitive and resistant cells in the absence or presence of ceritinib are shown in Table 1 and Table 2. Ceritinib produced a concentration-dependent decrease in the IC_50_ values of doxorubicin and paclitaxel (both are ABCB1 substrates) in KBv200 cells and MCF-7/adr cells but did not alter the cytotoxicity of cisplatin which is not an ABCB1 substrate. The similar results were observed in the stably transfected HEK293/ABCB1, but not in the parental sensitive cells, even at the maximum concentration used. Ceritinib also significantly enhanced the cytotoxicity of mitoxantrone and topotecan (ABCG2 substrate drugs) in ABCG2 overexpressing S1-M1-80 and transfected HEK293/ABCG2-R2 cells, while there was also no obvious effect on the parental S1 and HEK293/pcDNA3.1 cells. These results suggest that ceritinib enhances the sensitivity of ABCB1 and ABCG2 overexpressing MDR cells to conventional chemotherapeutic agents, but has negligible effect on their parental cell lines.

**Table 1:** Effect of Ceritinib on enhancement of conventional chemotherapeutic agents

	IC50 ± SD(μM) (fold-reversal)			
Compounds	KB		KBv200	
Doxorubicin	0.0145 ± 0.0023	(1.00)	0.9405 ± 0.0257	(1.00)
+ 0.125 μM Ceritinib	0.0135 ± 0.0003	(1.07)	0.4419 ± 0.0330**	(2.13)
+ 0.25 μM Ceritinib	0.0132 ± 0.0077	(1.10)	0.1937 ± 0.0230**	(4.86)
+ 0.5 μM Ceritinib	0.0130 ± 0.0058	(1.11)	0.1118 ± 0.0163**	(8.14)
+ 10 μM Verapamil	0.0159 ± 0.0103	(0.92)	0.0914 ± 0.0187**	(10.29)
Paclitaxel	0.0030 ± 0.0001	(1.00)	0.4315 ± 0.0066	(1.00)
+ 0.125 μM Ceritinib	0.0030 ± 0.0001	(1.00)	0.2886 ± 0.0037*	(1.50)
+ 0.25 μM Ceritinib	0.0030 ± 0.0001	(1.00)	0.1986 ± 0.0018**	(2.17)
+ 0.5 μM Ceritinib	0.0035 ± 0.0001	(0.86)	0.1301 ± 0.0021**	(3.32)
+ 10 μM Verapamil	0.0029 ± 0.0001	(1.03)	0.0285 ± 0.0035**	(15.14)
Cisplatin	1.3456 ± 0.1251	(1.00)	1.0838 ± 0.2131	(1.00)
+ 0.5 μM Ceritinib	1.1483 ± 0.1072	(1.17)	0.9690 ± 0.1373	(1.12)
	**MCF-7**		**MCF-7/adr(ABCB1)**	
Doxorubicin	0.1678 ± 0.0035	(1.00)	3.3674 ± 0.1310	(1.00)
+ 0.125 μM Ceritinib	0.1625 ± 0.0041	(1.03)	1.1301 ± 0.0031**	(2.98)
+ 0.25 μM Ceritinib	0.1957 ± 0.0029	(0.86)	0.9228 ± 0.0273**	(3.65)
+ 0.5 μM Ceritinib	0.1437 ± 0.0060	(1.17)	0.4697 ± 0.0321**	(7.17)
+ 10 μM Verapamil	0.1629 ± 0.0103	(1.03)	0.2371 ± 0.0274**	(14.2)
Paclitaxel	0.0233 ± 0.0003	(1.00)	0.4773 ± 0.0511	(1.00)
+ 0.125 μM Ceritinib	0.0232 ± 0.0042	(1.00)	0.2369 ± 0.0372**	(2.01)
+ 0.25 μM Ceritinib	0.0204 ± 0.0040	(1.14)	0.1653 ± 0.0214**	(2.89)
+ 0.5 μM Ceritinib	0.0229 ± 0.0037	(1.02)	0.0921 ± 0.0071**	(5.18)
+ 10 μM Verapamil	0.0195 ± 0.0006	(1.19)	0.0536 ± 0.0032**	(8.90)
Cisplatin	2.7325 ± 0.0373	(1.00)	24.2654 ± 0.2461	(1.00)
+ 0.5 μM Ceritinib	3.5385 ± 0.1352	(0.77)	21.3666 ± 0.4832	(1.14)
	**S1**		**S1-MI-80**	
Topotecan	0.2427 ± 0.0061	(1.00)	14.8718 ± 0.3460	(1.00)
+ 0.125 μM Ceritinib	0.2751 ± 0.0032	(0.88)	7.0011 ± 0.2131**	(2.12)
+ 0.25 μM Ceritinib	0.2100 ± 0.0041	(1.16)	4.6333 ± 0.1393**	(3.21)
+ 0.5 μM Ceritinib	0.1932 ± 0.0052	(1.26)	2.8469 ± 0.0794**	(5.22)
+ 2.5 FTC	0.2789 ± 0.0081	(0.87)	1.5424 ± 0.1320**	(9.64)
Mitoxantrone	0.1314 ± 0.0037	(1.00)	6.1269 ± 0.7233	(1.00)
+ 0.125 μM Ceritinib	0.1340 ± 0.0020	(0.98)	2.7024 ± 0.5381**	(2.27)
+ 0.25 μM Ceritinib	0.1251 ± 0.0056	(1.05)	1.3535 ± 0.0193**	(4.53)
+ 0.5 μM Ceritinib	0.1438 ± 0.0031	(0.91)	0.6479 ± 0.0932**	(9.46)
+ 2.5 μM FTC	0.1547 ± 0.0059	(0.85)	0.4846 ± 0.1083**	(12.64)
Cisplatin	6.7769 ± 0.1274	(1.00)	10.0453 ± 0.0260	(1.00)
+ 0.5 μM Ceritinib	6.5481 ± 0.0293	(1.03)	9.8959 ± 0.0373	(1.02)

Cell survival was performed by MTT assay as described in “Materials and Methods”. Data were shown as the mean ± standard deviation (SD) of at least three independent experiments performed in triplicate. The fold reversal of MDR (values given in parentheses) was calculated by dividing the IC_50_ value for cells with the anticancer agent in the absence of Ceritinib by that obtained in the presence of Ceritinib.

* *P* < 0.05.

** *P* < 0.01 significantly different from control group.

**Table 2:** Effect of ceritinib on reversing ABCB1- and ABCG2- mediated MDR in transfected cells

Compounds	IC50 ± SD(μM)		(fold-reversal)	
	HEK293/pcDNA3.1		HEK293/ABCB1	
Doxorubicin	0.0317 ± 0.0027	(1.00)	0.7258 ± 0.0263	(1.00)
+ 0.125 μM Ceritinib	0.0411 ± 0.0025	(0.77)	0.3351 ± 0.0371**	(2.17)
+ 0.25 μM Ceritinib	0.0338 ± 0.0003	(0.94)	0.2415 ± 0.0046**	(3.01)
+ 0.5 μM Ceritinib	0.0365 ± 0.0023	(0.87)	0.1246 ± 0.0041**	(5.82)
+ 10 μM Verapamil	0.0325 ± 0.0039	(0.98)	0.0421 ± 0.0032**	(17.22)
Paclitaxel	0.0215 ± 0.0023	(1.00)	1.2532 ± 0.1218	(1.00)
+ 0.125 μM Ceritinib	0.0173 ± 0.0037	(1.24	0.7683 ± 0.0753*	(1.63)
+ 0.25 μM Ceritinib	0.0161 ± 0.0031	(1.34)	0.4268 ± 0.0478**	(2.94)
+ 0.5 μM Ceritinib	0.0201 ± 0.0064	(1.07)	0.2306 ± 0.0349**	(5.44)
+ 10 μM Verapamil	0.0192 ± 0.0152	(1.12)	0.2048 ± 0.0437**	(6.12)
Cisplatin	0.9447 ± 0.1263	(1.00)	6.1129 ± 0.2135	(1.00)
+ 0.5 μM Ceritinib	0.6817 ± 0.1934	(1.39)	6.9635 ± 0.3172	(0.88)
	**HEK293/pcDNA3.1**		**HEK293/ABCG2-R2**	
Topotecan	0.0178 ± 0.0047	(1.00)	1.1756 ± 0.3182	(1.00)
+ 0.125 μM Ceritinib	0.0119 ± 0.0055	(1.50)	0.4143 ± 0.0643**	(2.84)
+ 0.25 μM Ceritinib	0.0180 ± 0.0003	(0.99)	0.2408 ± 0.0392**	(4.88)
+ 0.5 μM Ceritinib	0.0155 ± 0.0023	(1.14)	0.1025 ± 0.0094**	(11.45)
+ 2.5 μM FTC	0.0164 ± 0.0039	(1.09)	0.0874 ± 0.0031**	(13.46)
Mitoxantrone	0.1071 ± 0.0323	(1.00)	0.5731 ± 0.0731	(1.00)
+ 0.125 μM Ceritinib	0.0699 ± 0.0037	(1.53)	0.1389 ± 0.0393**	(4.13)
+ 0.25 μM Ceritinib	0.0724 ± 0.0429	(1.48)	0.0789 ± 0.0252**	(7.26)
+ 0.5 μM Ceritinib	0.0719 ± 0.0264	(1.49)	0.0341 ± 0.0271**	(16.80)
+ 2.5 μM FTC	0.0791 ± 0.0153	(1.35)	0.0214 ± 0.0380**	(26.81)
Cisplatin	0.9447 ± 0.1263	(1.00)	2.8384 ± 0.0152	(1.00)
+ 0.5 μM Ceritinib	0.6817 ± 0.0932	(1.39)	4.1839 ± 0.1973	(0.68)

Cell survival was performed by MTT assay as described in “Materials and Methods”. Data were shown as the mean ± standard deviation (SD) of at least three independent experiments performed in triplicate. The fold reversal of MDR (values given in parentheses) was calculated by dividing the IC_50_ value for cells with the anticancer drug in the absence of Ceritinib by that obtained in the presence of Ceritinib.

* *P* < 0.05.

** *P* < 0.01 significantly different from control group.

### Ceritinib enhanced the efficacy of conventional chemotherapeutic agents in ABCB1-mediated MDR in nude mouse xenografts

**Figure 2:**
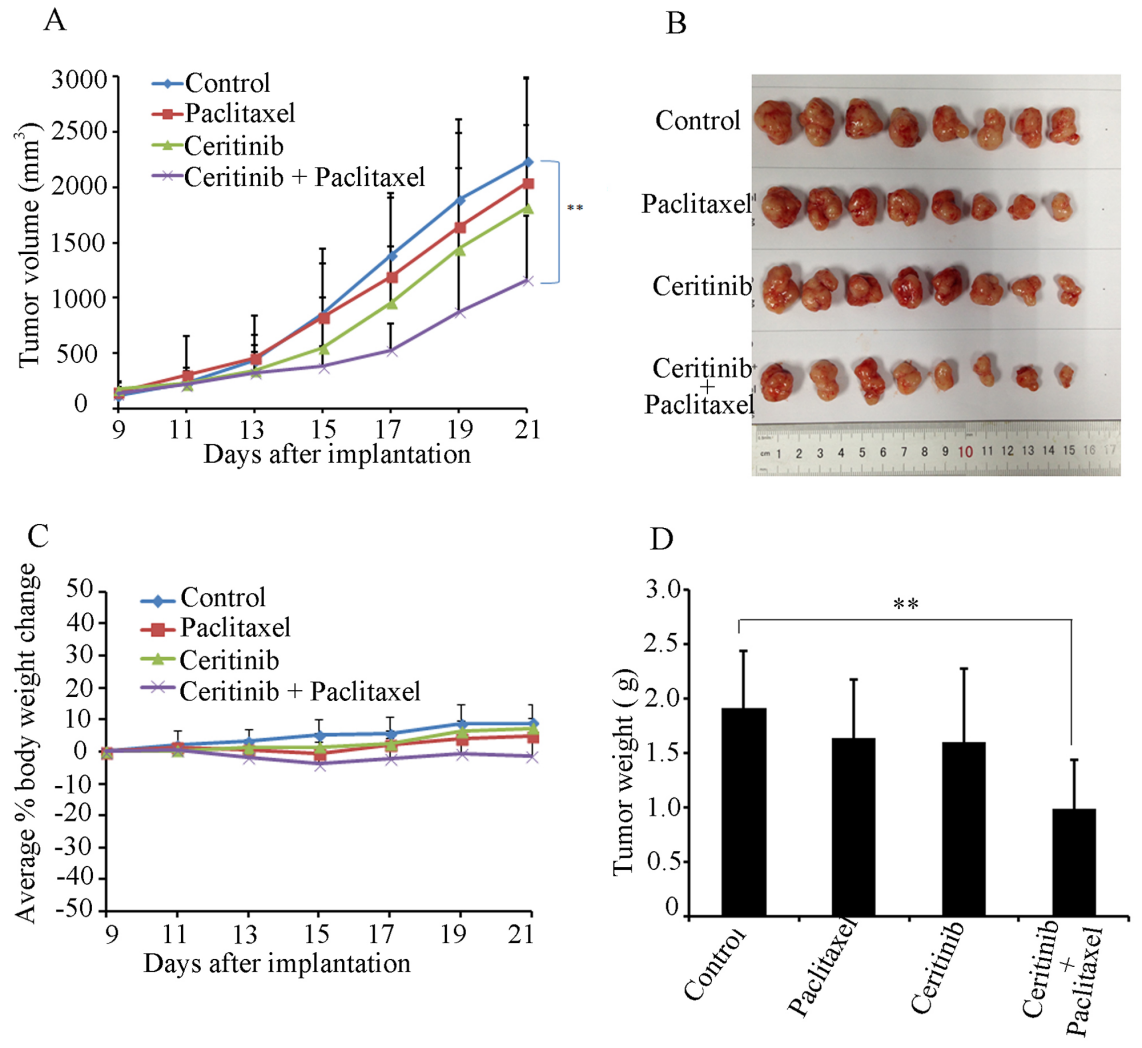
Ceritinib enhanced the anticancer effect of paclitaxel in the KBv200 cell xenograft model in nude mice **A.** the changes in tumor volume over time after the KBv200 cell implantation. Data shown are mean ± SD of tumor volumes for each group. *n* = 8. **B.** the image of tumors size in four groups excised from the mice on the 21th day after implantation. **C.** Average percentage change in body weight after treatments. **D.** mean tumor weight (*n* = 8) after excising from the mice on the 21th day after implantation. The four treatment groups were: (1) saline (q3d × 4); (2) paclitaxel (20 mg/kg, i.p., q3d × 4); (3) Ceritinib (25 mg/kg, p.o., q3d × 4); and (4) Ceritinib (25 mg/kg, p.o., q3d × 4 given 1 h before injecting paclitaxel) + paclitaxel (20 mg/kg, i.p., q3d × 4).

An ABCB1-mediated multidrug resistant KBv200 cell xenograft model was established in nude mice to evaluate whether ceritinib could reverse the resistance to paclitaxel *in vivo*. We did not find significant difference in tumor size between animals treated separately with saline, ceritinib or paclitaxel. However, there was a significant inhibition of tumor growth in the group with a combination of ceritinib and paclitaxel compared with other groups (*P* < 0.05; Figure 2). The mean weights of tumors excised from mice were 1.91 ± 0.52, 1.63 ± 0.54, 1.60 ± 0.66, 0.99 ± 0.44g for saline, paclitaxel, Ceritinib and combination group, respectively. Furthermore, we did not observe any death or apparent decrease in body weight in the combination treatment group at the doses tested, suggesting that the combination regimen did not increase toxicity.

### Ceritinib enhanced the accumulation of DOX and Rho123 in cells overexpressing ABCB1 and ABCG2

**Figure 3:**
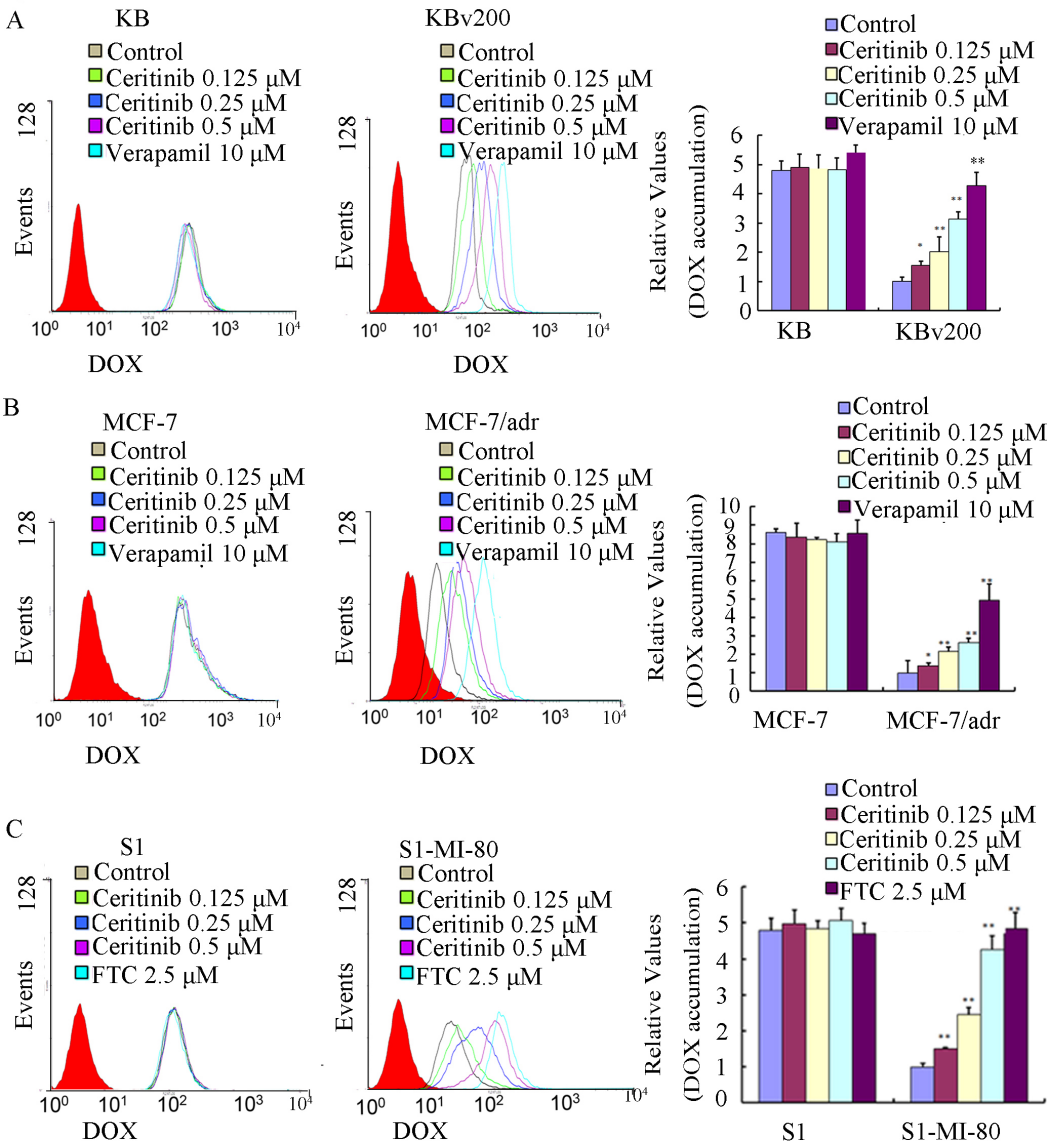
Effect of ceritinib on the intracellular accumulation of Dox in MDR cells and their parental cells The accumulation of DOX **A, B, C.** in KBv200, MCF-7/adr, S1-MI-80 cells and their parental cells were measured by flow cytometric analysis as described in Materials and Methods, The results were presented as fold change in fluorescence intensity relative to control MDR cells. Columns, means of triplicate determinations; bars, SD. “*” *P* < 0.05, “**” *P* < 0.01 significantly different from control group.

**Figure 4:**
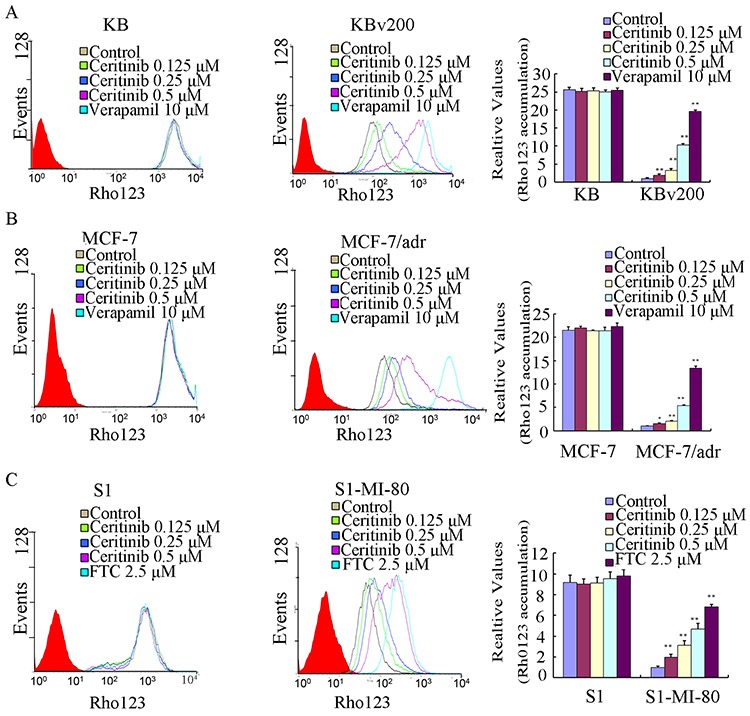
Effect of ceritinib on the intracellular accumulation of Rho123 in MDR cells and their parental cells The accumulation of Rho123 **A, B, C.** in KBv200, MCF-7/adr, S1-MI-80 cells and their parental cells were measured by flow cytometric analysis as described in Materials and Methods, The results were presented as fold change in fluorescence intensity relative to control MDR cells. Columns, means of triplicate determinations; bars, SD. “*” *P* < 0.05, “**” *P* < 0.01 significantly different from control group.

The results described above revealed that ceritinib could enhance the sensitivity of ABCB1 and ABCG2-overexpressing cells to the transporter substrate anticancer agents *in vitro* and *in vivo*. To investigate the potential mechanisms for the MDR reversal by ceritinib, we examined the intracellular accumulation of DOX and Rho 123 in the presence or absence of ceritinib in ABCB1- and ABCG2- overexpressing MDR cells and their parental cells. Our results showed that the intracellular accumulation of DOX or Rho123 in resistant cells was significantly lower than that in sensitive cells in the absence of ceritinib. Importantly, ceritinib was found to significantly increase the intracellular accumulation of DOX (fluorescent substrate drug of both ABCB1 and ABCG2) and Rho123 (fluorescent probe substrate of ABCB1 and ABCG2) in a concentration-dependent manner in KBv200, MCF-7/adr and S1-MI-80, while no notable change was observed in the parental cells (Figure 3 & 4). These results suggested that ceritinib could increase intracellular accumulation of chemotherapeutic agents in ABCB1 and ABCG2-overexpressing cells.

### Ceritinib inhibited the efflux of DOX in MDR cells overexpressing ABCB1

**Figure 5:**
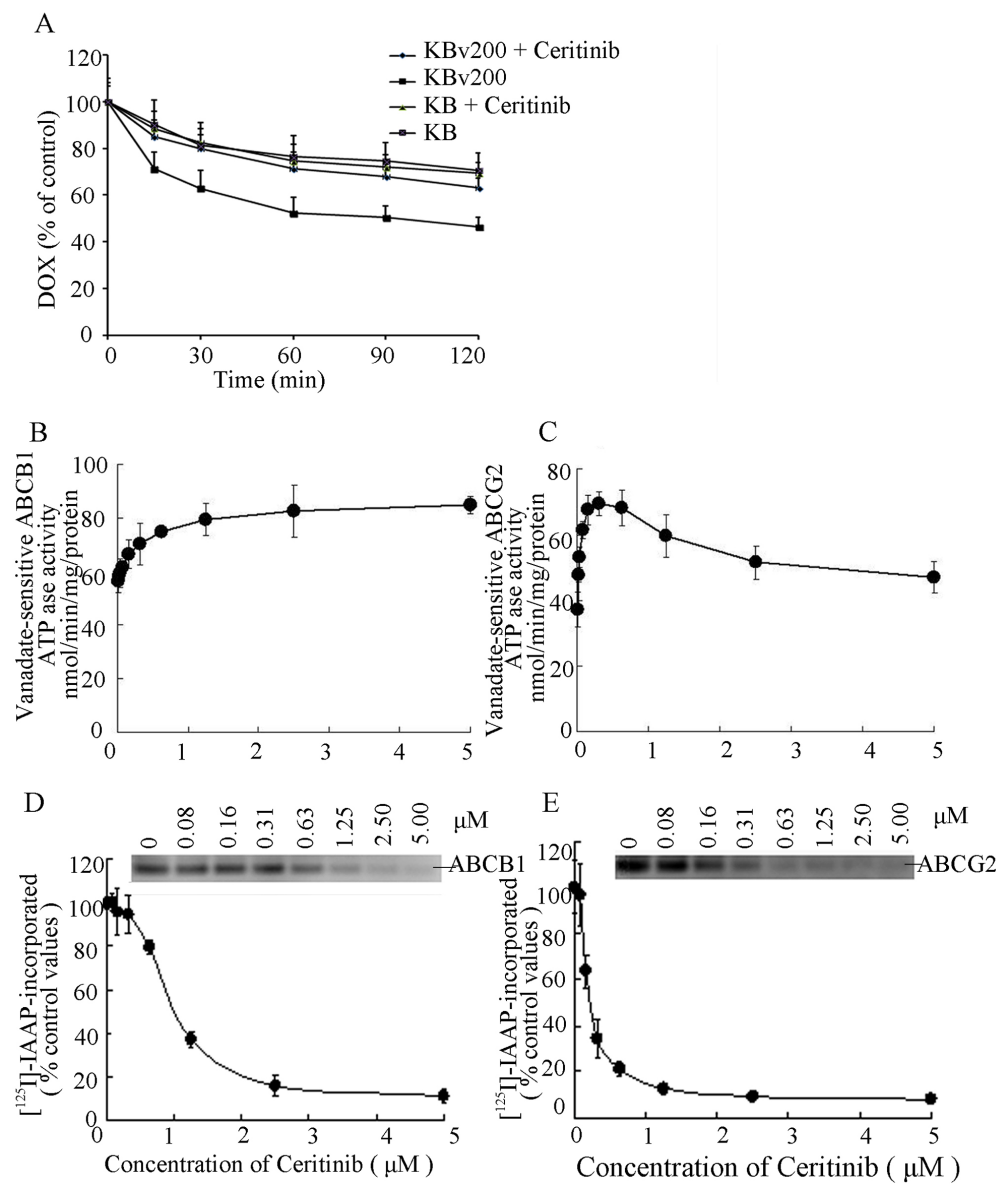
Effect of ceritinib on the efflux of DOX, the ATPase activity of ABCB1 and ABCG2 and the [125I]-IAAP photoaffinity labeling of ABCB1 and ABCG2 **A.** Time course of Dox efflux was measured in KB and KBv200 cells, with or without 0.5 μM Ceritinib. **B, C.** Effect of ceritinib on ATPase activity of ABCB1 and ABCG2. The vanadate-sensitive ABCB1 or ABCG2 ATPase activity in the presence of the indicated concentrations of ceritinib was evaluated. The mean and standard error values from three independent experiments are shown. **D, E.** Ceritinib competed for photolabeling of ABCB1 or ABCG2 by [^125^I]-IAAP. Crude membranes from High Five insect cells expressing ABCB1 or ABCG2 were incubated with [^125^I]-IAAP and increasing concentration (0 – 5 μM) of ceritinib. The samples were then cross-linked by UV illumination, subjected to electrophoresis, and analyzed as outlined under Materials and Methods. A representative autoradiogram from three independent experiments is shown. The relative amount of [^125^I]-IAAP incorporated is plotted against the concentration of ceritinib present. 100% incorporation refers to the absence of ceritinib.

Ceritinib increased intracellular accumulation of DOX and Rho123 in ABCB1-overexpression MDR cells; Next, we examined whether the increased accumulation of anticancer agents was due to inhibition of efflux of anticancer agents. The efflux of DOX over 2 h after an initial drug accumulation was monitored and the result is shown in Figure 5A. As expected, due to ABCB1 overexpression in KBv200 cells, DOX retention dropped remarkably from 100% (0 h efflux) to about 46.4% (2 h efflux). The decrease in DOX retention was much less in the parental KB cells (69.4% retention at 2 h). Importantly, ceritinib (0.5 μM) was found to significantly increase DOX retention (*p* < 0.05) in KBv200 cells to 63.0% of the level attained at the 2 h time point. The result shows that ceritinib inhibited drug efflux of ABCB1 in KBv200 cells but did not influence drug efflux in sensitive KB cells.

### Ceritinib stimulated the ATPase activity of ABCB1 and ABCG2

The drug-efflux function of ABCB1 and ABCG2 is linked to ATP hydrolysis which is stimulated in the presence of ABCB1 and ABCG2 substrates. To understand whether ceritinib influenced the ATPase activity of ABCB1 and ABCG2, we measured the vanadate-sensitive ATPase activity of ABCB1 and ABCG2 in the presence of a range of different concentrations of ceritinib. While ceritinib activated the ATPase activity of ABCB1 in a concentration-dependent manner, it stimulated the ATPase activity of ABCG2 at low concentrations but the stimulated ABCG2 ATPase activity dropped at higher concentration of ceritinib (Figure 5B, 5C). These results indicated that ceritinib may be a substrate of ABCB1 and ABCG2.

### Ceritinib bound to substrate binding site of ABCB1 and ABCG2

The substrate binding site of ABCB1 or ABCG2 could be photo-labeled by [^125^I]-IAAP. It is known that substrate of ABCB1 or ABCG2 could compete for [^125^I]-IAAP labeling of the two transporters [12]. To further ascertain the interaction of ceritinib with the substrate binding sites of ABCB1 and ABCG2, we examined the photo-labeling of ABCB1 and ABCG2 with [^125^I]-IAAP by incubating membrane vesicles in the presence of various concentrations of ceritinib. As showed in (Figure 5D, 5E), ceritinib strongly inhibited the photoaffinity labeling of ABCB1and ABCG2 with [^125^I]-IAAP in a concentration-dependent manner. The results suggest that ceritinib binds with high affinity to both the ABCB1 and ABCG2 substrate-binding sites.

### Ceritinib did not alter the expression level of ABCB1 and ABCG2

**Figure 6:**
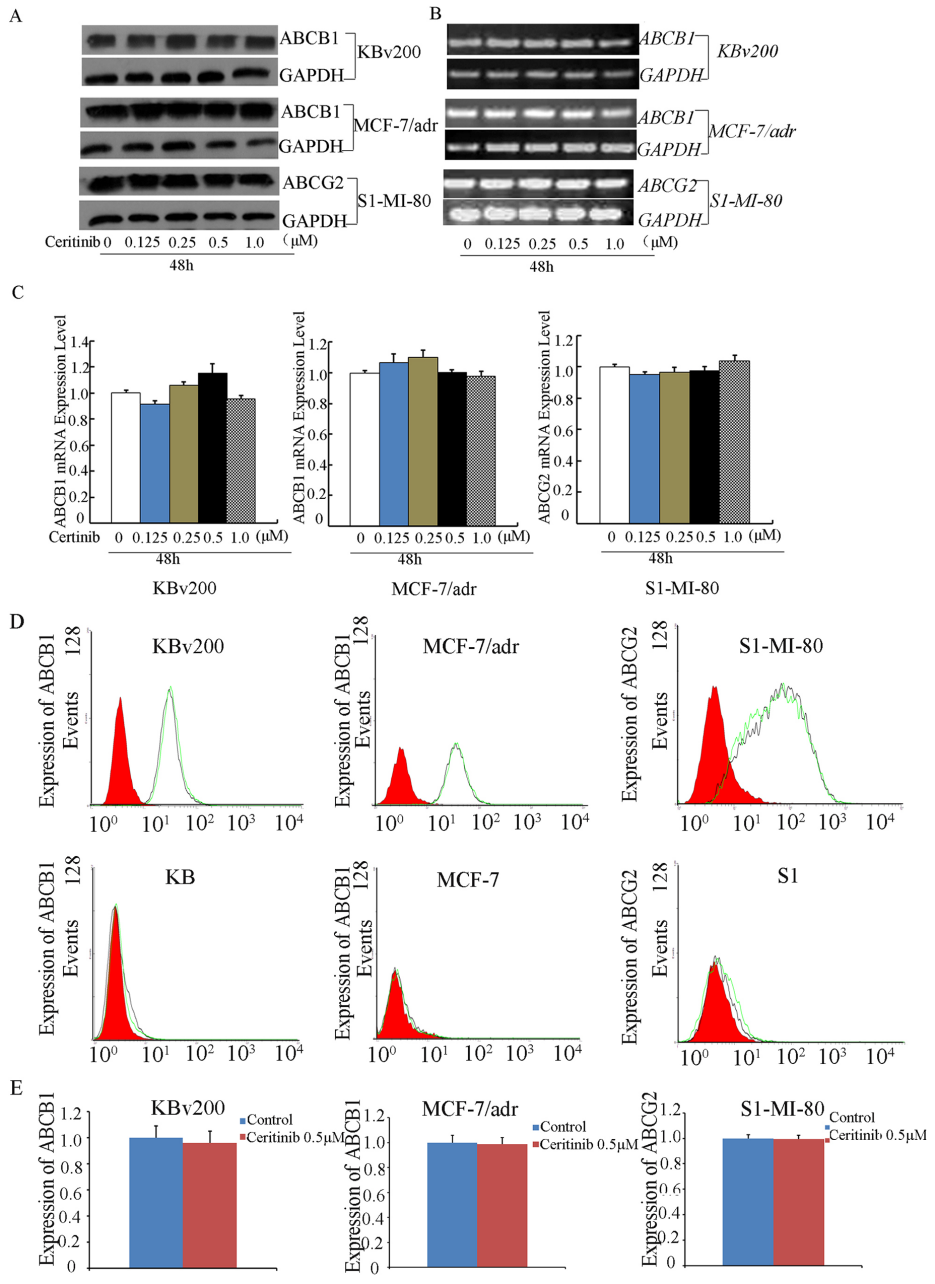
Effect of ceritinib on the expression of ABCB1 or ABCG2 in cells **A.** The protein level of ABCB1 and ABCG2 were measured by Western blot analysis, and **B.** mRNA level were measured by RT-PCR, Ceritinib did not alter the mRNA and protein levels in MDR cells. **C.** q-PCR was further applied to confirm the unaltered mRNA levels in MDR cells. **D, E.** the cell surface expression of ABCB1 and ABCG2 were measured by Flow Cytometer. All these experiments were repeated at least three times, and data from a representative experiment is shown in each panel.

ABC transporter-mediated MDR may be circumvented by downregulating the expression of the transporters or by inhibiting their transport function. Therefore, we determined the effect of ceritinib on the mRNA and protein expression of ABCB1 and ABCG2 using RT-PCR and Western blot analysis, respectively. Our results showed that no significant difference in ABCB1 or ABCG2 expression at the mRNA or protein level was observed in KBv200, MCF-7/adr cells or S1-M1-80 cells treated with 0.125, 0.25, 0.5 μM ceritinib for 48 h compared to untreated control cells (Figure 6A–6C). Therefore, it is likely that ceritinib reversed MDR by inhibiting the transport function of ABCB1 and ABCG2, but not by downregulating the expression of the transporters.

### Ceritinib did not change the cell surface expression of ABCB1 and ABCG2

To further understand whether the localization of ABCB1 and ABCG2 were influenced by ceritinib, we analyzed the cell surface expression of ABCB1 and ABCG2 in the presence or absence of 0.5 μM ceritinib in ABCB1- or ABCG2- overexpressing MDR cells and their parental cells. We found that the expression of ABCB1 or ABCG2 was no obvious change in KBv200, MCF-7/adr and S1-MI-80 in the presence or absence of 0.5 μM ceritinib, and their parental cells no expression of ABCB1 or ABCG2, respectively (Figure 6D, 6E). These suggest ceritinib did not alter the localization of ABCB1 or ABCG2.

### Ceritinib had no effect on the blockage of AKT and ERK1/2 phosphorylation at its MDR reversal concentrations

**Figure 7:**
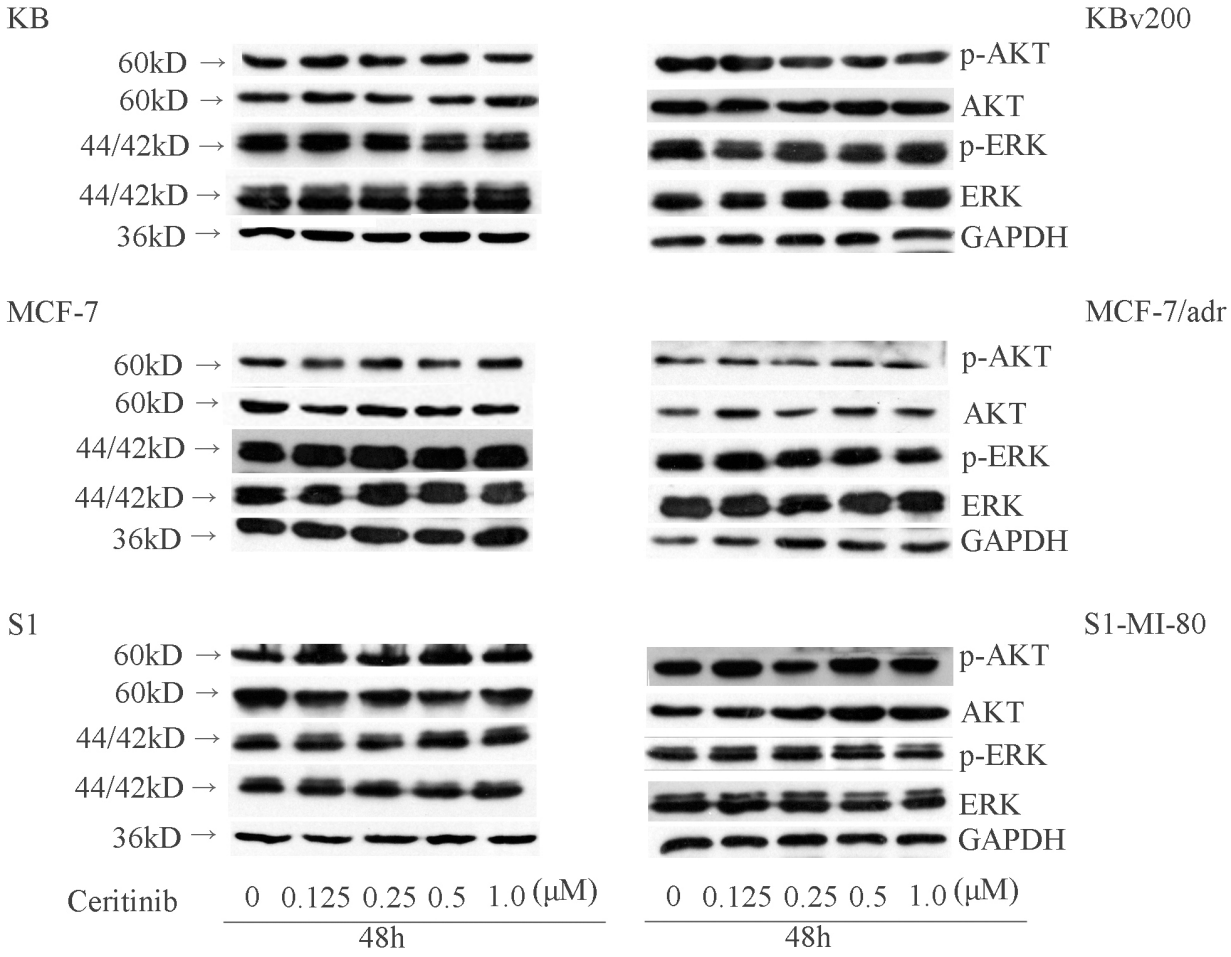
Effect of ceritinib on the phosphorylation of AKT and ERK1/2 KBv200, MCF-7/adr and S1-MI-80 and their parental cells were treated with different concentrations of ceritinib for 48 h. Equal amount of protein was loaded for Western blot analysis as described in “Materials and Methods”. Independent experiments were performed at least three times.

Previous studies have shown that inhibiting the Akt and ERK1/2 pathways may decrease the resistance to anticancer drugs in cancer cells [13, 14]. To determine whether the enhancement effect of ceritinib was related to the change in phosphorylation status of AKT and ERK1/2 in KBv200, MCF-7/adr, S1-MI-80 and their parental cells, we examined the total and phosphorylation of AKT and ERK1/2 after treating the cells with 0.125, 0.25, 0.5 μM ceritinib for 48 h. As shown in Figure 7, there was no significant alteration in the total and phosphorylated forms of AKT or ERK1/2. These results suggested that the enhancement effect of ceritinib on ABCB1 and ABCG2 overexpressing cells is independent of the inhibition of AKT and ERK1/2 phosphorylation.

## DISCUSSION AND CONCLUSIONS

Multidrug resistance is the major obstacle to the successful chemotherapy of cancer. A number of different mechanisms are known to contribute to drug resistance, which include altered cell cycle check points, inhibition of apoptosis, activation of DNA repair, decreased uptake and increased efflux of anticancer drugs. The overexpression of ABC transporters is probably one of the earliest event when a cancer cell acquire multidrug resistance and it is also the most commonly reported. The broad substrate specificity of MDR transporters implied that the drug resistance can affect simultaneously a broad spectrum of anticancer drugs with diverse structure and functional features. The recent discovery of potent and specific inhibition of various ABC transporters by TKIs represents a new strategy to circumvent MDR. To this end, TKIs have been reported to interact with the MDR transporters and inhibit the transport activity by competitive inhibition. For instance, Imatinib (STI-571) was found to be an ABCB1 substrate [15], and EKI-785 was shown to interact with ABCC1 [16], Moreover, CI1033 was reported to be substrates and inhibitors of ABCG2 [17, 18]. Other TKIs such as gefitinib, erlotinib, vandetanib, lapatinib and apatinib have also been shown to inhibit ABCB1 and ABCG2 function [19–24].

Ceritinib is a promising TKI targeting ALK and it is currently in phase III clinical trial [10]. In the clinic, ceritinib is indicated to treat patients with ALK-positive, metastatic lung cancer with disease progression or who cannot tolerate crizotinib [25]. Importantly, ceritinib was also shown to be effective in patients not responding to crizotinib [11], Our results showed for the first time that ceritinib potently reversed both ABCB1- and ABCG2-mediated MDR. The MDR reversal effect is specific because ceritinib did not affect the cytotoxicity of the concomitantly administered anticancer drugs in the parental sensitive cells (KB, MCF-7 & S1) and the mock transfected (HEK293/pcDNA3.1) cells. Moreover, ceritinib also did not affect the cytotoxicity of cisplatin (a non-substrate of ABCB1 and ABCG2).

To elucidate the mechanism of MDR reversal, we evaluated the inhibition of substrate drug efflux by ceritinib in the ABCB1 and ABCG2-overepressing cancer cells. Consistent with the drug sensitization observed in the resistant cells, ceritinib was found to inhibit the efflux function of both ABCB1 and ABCG2, thereby increasing the accumulation of anticancer drugs in the cells.

ABC transporters exert their drug efflux function by utilizing energy derived from the hydrolysis of ATP to transport their substrates across the membrane against a concentration gradient [26]. Therefore, the rate of ATP hydrolysis (ATPase activity) is related to the transport activity of ABC transporters. Some TKIs such as lapatinib [27], sunitinib [28], erlotinib [20], apatinib [29], crizotinib [30], even at low concentrations could stimulate the ATPase activities of the transporters. We found ceritinib stimulated both vanadate-sensitive ABCB1 and ABCG2 ATPase. These results suggested that ceritinib is likely a substrate of both ABCB1 and ABCG2. This is further confirmed by the observation that ceritinib competed with [^125^I]-IAAP for the photoaffinity labeling of the two transporters.

The reversal of MDR may be achieved by downregulation of the ABC transporter expression. To this end, we found that ceritinib did not alter the expression of ABCB1 and ABCG2 at the MDR reversal concentration. Ceritinib is an inhibitor of ALK tyrosine kinase. Preclinical studies demonstrated that ceritinib can led to suppression of ALK phosphorylation as well as the downstream PI3K/AKT, MEK/ERK and mTOR signaling pathways[31, 32]. It was reported that the blockage of phosphorylation of PI3K/AKT and/or ERK pathways could enhance the efficacy of chemotherapeutic agents [32] We found that ceritinib did not alter the expression and phosphorylation level of AKT and ERK1/2. These demonstrated the reversal of MDR by ceritinib was not associated with the inhibition AKT and ERK pathways.

Taken together, our results advocate the further investigation of the combination of ceritinib with other conventional chemotherapeutic drugs in the clinic for the treatment of patients with ABCB1 or ABCG2-mediated MDR.

## MATERIALS AND METHODS

### Materials

Dulbecco's modified Eagle medium (DMEM) and RPMI-1640 were obtained from Gibco BRL (Thermo Fisher Scientific Inc., Waltham, MA, USA). Doxorubicin, paclitaxel, cisplatin, topotecan, mitoxantrone, Verapamil (VRP), Fumitremorgin C (FTC), rhodamine123, 3-(4,5-dimethylthiazol-2-yl)-2,5-diphenyltetrazolium bromide (MTT) and dimethyl sulfoxide (DMSO) were all purchased from Sigma-Aldrich (St. Louis, MO, USA). Ceritinib was purchased from selleck chemicals (Houston, TX, USA), with a molecular structure as shown in Figure 1A. Monoclonal antibodies against ABCB1, ABCG2, extracellular signal-regulated protein kinases 1 and 2 (ERK1/2), p-ERK, AKT and p-AKT were from Santa Cruz Biotechnology (Santa Cruz, CA, USA). The antibody against glyceraldehyd-3-Phosphate dehydrogenase (GAPDH) was from Kangcheng (Shanghai, China).

### Cell culture

The human oral epidermoid carcinoma cell line KB and its vincristine-selected ABCB1 overexpressing derivative KBv200 cells [33]; the human breast carcinoma cell line MCF-7, its doxorubicin selected ABCB1 overexpressing derivative MCF-7/adr cells [34]; the human colon carcinoma cell line S1 and its mitoxantrone-selected ABCG2-overexpressing derivative S1-M1-80 cells [35] and the human embryonic kidney cell line HEK293 and its stable pcDNA3.1, ABCB1 and ABCG2 stable gene transfect cell lines HEK293/pcDNA3.1, HEK293/ABCB1, HEK293/ABCG2-R2 were kind gift provided by Dr. Susan Bates (National Cancer Institute, NIH, Bethesda, MD, U.S.A) [36]. The transfected cells were cultured in medium containing 2 mg/mL G418. All cell lines were cultured in DMEM or RPMI 1640 supplemented with 10% FBS at 37°C in a humidified atmosphere of 5% CO2. All cells were grown in drug-free culture medium for more than 2 weeks before assay.

### Cell cytotoxicity assay

The MTT assay was used to assess cytotoxicity as described previously [37]. Briefly, cells growing in logarithmic phase were seeded at a density of 2000 ~ 6000 cells per well in 96-well plates. When the cells became adherent 24 h later, a range of different concentrations of conventional chemotherapeutic drugs with or without a fixed combination of ceritinib were added to the wells. After 68 h, MTT (5 mg/ml, 20 μL) was added into each well, and 4 h later, the medium was discarded and 150 μL DMSO was added into the wells to dissolve the formazan product from the metabolism of MTT. Finally, optical density was measured at 540 nm, with background subtraction at 670 nm by a Model 550 Microplate Reader (Bio-Rad, Hercules, CA, USA). The half maximal (50%) inhibitory concentration (IC_50_) value of a substance was calculated by the Bliss method [38]. The fold reversal of MDR was calculated as previously described [29]. All experiments were repeated at least three times.

### Establishment of KBv200 cell xenograft model and reversal of MDR by ceritinib *in vivo*

The KBv200 cell xenograft model was established as described previously with minor modification [39], Athymic nude mice (5–6 weeks old) were purchased from the center of experimental animals (Southern Medical University). Briefly, KBv200 cells grown *in vitro* were harvested and implanted subcutaneously under the shoulder in the nude mice. When the tumors reached a mean diameter of 0.5 cm, the mice were randomized into four groups and treated as follows: (1) saline (q3d × 4); (2) paclitaxel (20 mg/kg, i.p., q3d × 4); (3) Ceritinib (25 mg/kg, p.o., q3d × 4); and (4) Ceritinib (25 mg/kg, p.o., q3d × 4 given 1 h before injecting paclitaxel)+ paclitaxel (20 mg/kg, i.p., q3d × 4). The body weights of the animals and the two perpendicular diameters (A and B) were recorded every 2 days, and tumor volume (V) was estimated according to the following formula:

$$\text{V}=\frac{\pi }{6}{\left(\frac{\text{A}+\text{B}}{2}\right)}^{3}$$

The curve of tumor growth was drawn according to tumor volume and time of implantation. The mice were anesthetized and sacrificed when the mean of tumor weights was over 1 g in the control group. Tumor tissues were excised from the mice and their weight was measured. The ratio of growth inhibition (IR) was calculated according to the following formula:

$$\text{IR}(\%)=1-\frac{\text{Mean tumor weight of experimental group}}{\text{Mean tumor weight of control group}\;}\times 100\%$$

### Doxorubicin (DOX) and rhodamine 123 (rho 123) accumulation

The effect of ceritinib on the accumulation of DOX and Rho 123 was measured by flow cytometry as previously described [40]. Briefly, the cells were incubated in six-well plates to allow attach to the wall overnight. Then the cells were exposed to different concentrations of ceritinib (0.125, 0.25, 0.5 μM). After 3 h, DOX (10 μM) or Rho 123 (5 μM) was added to the medium for further incubation for another 3 h or 0.5 h, then the cells were collected, centrifuged and washed three times with ice-cold phosphate-buffered saline (PBS) buffer, cells were resuspended in 500 μl PBS buffer for flow-cytometric analysis (Cytomics FC500; Beckman Coulter Inc., Brea, CA, USA). Verapamil, a known ABCB1 inhibitor, was used as a positive control for KBv200, KB, MCF-7/adr, MCF-7 cells [41]. Fumitremorgin C (FTC), a specific ABCG2 inhibitor, was used as a positive control of ABCG2 in S1-MI-80 and S1 cells [42].

### DOX efflux test

DOX efflux was performed following a modification of methods described earlier [28]. KB and KBv200 cells were treated with 10 μM DOX for 3 h at 37°C, then the cells were washed three times and subsequently maintained at 37°C with culture media without DOX in the presence or absence of 0.5 μM ceritinib at 0, 15, 30, 60 and 120 min, cells were collected and washed three times with ice-cold PBS. Finally, cells were resuspended in ice-cold PBS buffer for flow cytometric analysis immediately (Beckman Coulter).

### ABCB1 and ABCG2 ATPase activity assay

A colorimetric ATPase assay was performed as previously described with minor modification [43]. Briefly, crude membranes isolated from High Five insect cells expressing either ABCB1 or ABCG2 (100 μg protein/mL) were incubated at 37°C with a range of different concentrations of ceritinib in the presence or absence of sodium orthovanadate (0.3 mM for ABCB1 and 1.2 mM for ABCG2) in ATPase assay buffer (50 mM KCl, 5 mM sodium azide, 2 mM EDTA, 10 mM MgCl_2_, 1 mM DTT, pH 6.8) for 5 min. The crude membranes were kind gift provided by Dr Suresh Ambudkar (National Cancer Institute, NIH, USA). ATP hydrolysis reaction was then started by the addition of 5 mM Mg-ATP (concentration in a final volume of 60 μL) and incubated for 20 min (for ABCB1) or 10 min (for ABCG2). SDS solution (30 μL of 10% SDS) was added to terminate the reaction. Absorbance was subsequently measured at 750 nm after the addition of a detection reagent (35 mM ammonium molybdate, 15 mM zinc acetate, 10% ascorbic acid) and incubation at 37°C for 20 min. The amount of inorganic phosphate released was quantified by reading from a standard curve. Specific ceritinib-stimulated ABCB1 and ABCG2 ATPase activity (i.e. vanadate-sensitive) was determined as the difference between the amounts of inorganic phosphate released from ATP in the absence and presence of sodium orthovanadate.

### Photo-affinity labeling of ABCB1 and ABCG2 with [^125^I]-IAAP

Crude membrane from High Five insect cells expressing ABCB1 or ABCG2 (50 μg protein) was incubated with 0 – 5 μM ceritinib for 5 min at room temperature in 50 mM Tris-HCl (pH 7.5). [^125^I]-IAAP (2200 Ci/nmole, 3 nM) was added and incubation was continued for a further 5 min under subdued light. The samples were then cross-linked by UV illumination (365 nm) on ice. The labeled ABCB1 and ABCG2 was immunoprecipitated using the C219 and BXP21 antibody, respectively. The samples were then subjected to SDS-PAGE using a 7% Tris-acetate NuPAGE gel, dried and exposed to Bio-Max MR film (Eastman Kodak Co., Rochester, NY) at −80°C for 4 h. The radioactivity incorporated into the transporter protein was quantified using the Storm 860 PhosphorImager system (Molecular Dynamics, Sunnyvale, CA).

### Reverse transcription-polymerase chain reaction (PCR) and Q-PCR

ABCB1 and ABCG2 mRNA expression level was assayed as previously described [27]. Cells were treated with different concentrations of ceritinib for 48 h, after which total cellular RNA was isolated by Trizol Reagent RNA extraction kit following the manufacturer's instruction (Molecular Research Center, Cincinnati, OH, USA). The first strand cDNA was synthesized by Oligo dT primers with reverse transcriptase (Promega Corp.). PCR primers were 5′-CCCATCATTGCAATAGCAGG-3′ (forward) and 5′-GTTCAAACTTCTGCTCCTGA-3′ (reverse) for ABCB1, 5′-TGGCTGTCATGGCTTCAGTA-3′ (forward) and 5′-GCCACGTGATTCTTCCACAA-3′ (reverse) for ABCG2, 5′-CTTTGGTATCGTGGAAGGA-3′ (forward) and 5′-CACCCTGTTGCTGTAGCC-3′ (reverse) for GAPDH, reactions were carried out at 94°C for 2 min for initial denaturation, and then at 94°C for 30 s, 58°C for 30 s and 72°C for 1 min. After 35 cycles of amplification, additional extensions were carried out at 72°C for 10 min. At last products were resolved and examined by 1% agarose gel electrophoresis.

Real-time PCR was performed with Real-time PCR Master Mix containing SYBR GREEN I and hotstart Taq DNA polymerase. SYBR Green Assay kit (Molecular Research Center, Cincinnati, OH, USA) was used for real time PCR reaction, following manufacturer's protocol. The reaction was under the following conditions: 50°C for 2 min, 95°C for 5 min and 40 cycles at 95°C for 15 s, 60°C for 30 s. Data were analyzed using the 2−ΔΔCt method and normalized by GAPDH expression in each sample.

### Western blot analysis

The protein expression of ABCB1, ABCG2 or the phosphorylation of AKT and ERK1/2 were evaluated in the ABCB1 and ABCG2 over-expressing cells and their parental cells after treatment with ceritinib (0.125, 0.25 and 0.5 μM) for 48 h. Western blot analysis was conducted as previously described [27]. After blocking with 5% nonfat milk for 2 hours at room temperature, the membranes were immunoblotted by using antibodies including ABCB1, ABCG2, ERK1/2, p-ERK, AKT and p-AKT overnight at 4°C. The membranes were then washed three times with TBST and incubated with HRP-conjugated secondary antibody at 1:5000 dilution for 2 h at room temperature. After three times washed with TBST, the protein-antibody complexes were visualized by the enhanced Phototope TM-HRP Detection Kit (Cell Signaling) and exposed to Kodak medical X-ray processor (Carestream Health, Atlanta, GA, USA). GAPDH was used as a loading control.

### Detection of cell surface expression of ABCB1 and ABCG2 by flow cytometer

KBv200, KB, MCF-7/adr, MCF-7, S1-MI-80 and S1 cells were collected and washed three times with an isotonic PBS buffer (supplemented with 0.5% bovine serum albumin(BSA)). For ABCB1 expression analysis, approximately 1 × 10^6^ KBv200, KB, MCF-7/adr and MCF-7 cells (100ul) were incubated at 4°C for 45 min with 10 μL of FITC-conjugated anti-human pgp (Beckman Coulter, Fullerton, CA, USA), then the cells were washed twice with PBS buffer (supplemented with 0.5% BSA) and resuspended in 400 μL PBS buffer for flow cytometric analysis, Isotype control samples were treated with mouse IgG2a antibody in parallel For ABCG2 expression analysis, FITC-conjugated anti-human Bcrp1/*ABCG2* (Santa Cruz, CA, USA) reagent were mixed with 25 μL of Fc-blocked cells (1 × 10^6^ cells). After incubating for 45 min at 4°C, the cells were washed twice with PBS buffer (supplemented with 0.5% BSA) and resuspended in 400 μL PBS buffer for flow cytometric analysis, Isotype control samples were treated in an identical manner with FITC-labeled mouse immunoglobin G2b (IgG2b) antibody. All experiments were repeated at least three times.

### Data analysis

All experiments were repeated at least three times, and the results were showed as mean values ± standard deviation (SD). The statistical software SPSS16.0 was used in data processing and analysis. Statistical significance was determined at *P* < 0.05 or *P* < 0.01 by the Student's *t*-test.

## References

[1] Szakacs G, Paterson JK, Ludwig JA, Booth-Genthe C, Gottesman MM (2006). Targeting multidrug resistance in cancer. Nat Rev Drug Discov.

[2] Vasiliou V, Vasiliou K, Nebert DW (2009). Human ATP-binding cassette (ABC) transporter family. Hum Genomics.

[3] Ambudkar SV, Dey S, Hrycyna CA, Ramachandra M, Pastan I, Gottesman MM (1999). Biochemical, cellular, and pharmacological aspects of the multidrug transporter. Annu Rev Pharmacol Toxicol.

[4] Gottesman MM, Ling V (2006). The molecular basis of multidrug resistance in cancer: the early years of P-glycoprotein research. FEBS Lett.

[5] Miyake K, Mickley L, Litman T, Zhan Z, Robey R, Cristensen B, Brangi M, Greenberger L, Dean M, Fojo T, Bates SE (1999). Molecular cloning of cDNAs which are highly overexpressed in mitoxantrone-resistant cells: demonstration of homology to ABC transport genes. Cancer Res.

[6] Mao Q, Unadkat JD (2005). Role of the breast cancer resistance protein (ABCG2) in drug transport. AAPS J.

[7] Krishnamurthy P, Schuetz JD (2006). Role of ABCG2/BCRP in biology and medicine. Annu Rev Pharmacol Toxicol.

[8] Cole SP, Bhardwaj G, Gerlach JH, Mackie JE, Grant CE, Almquist KC, Stewart AJ, Kurz EU, Duncan AM, Deeley RG (1992). Overexpression of a transporter gene in a multidrug-resistant human lung cancer cell line. Science.

[9] McGrath T, Latoud C, Arnold ST, Safa AR, Felsted RL, Center MS (1989). Mechanisms of multidrug resistance in HL60 cells. Analysis of resistance associated membrane proteins and levels of mdr gene expression. Biochem Pharamcol.

[10] Marsilje TH, Pei W, Chen B, Lu W, Uno T, Jin Y, Jiang T, Kim S, Li N, Warmuth M, Sarkisova Y, Sun F, Steffy A, Pferdekamper AC, Li AG, Joseph SB (2013). Synthesis, structure-activity relationships, and *in vivo* efficacy of the novel potent and selective anaplastic lymphoma kinase (ALK) inhibitor 5-chloro-N2-(2-isopropoxy-5-methyl-4-(piperidin-4-yl)phenyl)-N4-(2-(isopropylsulf onyl)phenyl)pyrimidine-2,4-diamine (LDK378) currently in phase 1 and phase 2 clinical trials. J Med Chem.

[11] Friboulet L, Li N, Katayama R, Lee CC, Gainor JF, Crystal AS, Michellys PY, Awad MM, Yanagitani N, Kim S, Pferdekamper AC, Li J, Kasibhatla S, Sun F, Sun X, Hua S (2014). The ALK inhibitor ceritinib overcomes crizotinib resistance in non-small cell lung cancer. Cancer Discov.

[12] Shukla S, Robey RW, Bates SE, Ambudkar SV (2006). The calcium channel blockers, 1,4-dihydropyridines, are substrates of the multidrug resistance-linked ABC drug transporter, ABCG2. Biochemistry-US.

[13] Oh SY, Song JH, Gil JE, Kim JH, Yeom YI, Moon EY (2006). ERK activation by thymosin-beta-4 (TB4) overexpression induces paclitaxel-resistance. Exp Cell Res.

[14] Gagnon V, Van Themsche C, Turner S, Leblanc V, Asselin E (2008). Akt and XIAP regulate the sensitivity of human uterine cancer cells to cisplatin, doxorubicin and taxol. Apoptosis.

[15] Burger H, van Tol H, Boersma AW, Brok M, Wiemer EA, Stoter G, Nooter K (2004). Imatinib mesylate (STI571) is a substrate for the breast cancer resistance protein (BCRP)/ABCG2 drug pump. Blood.

[16] Hegedus T, Orfi L, Seprodi A, Varadi A, Sarkadi B, Keri G (2002). Interaction of tyrosine kinase inhibitors with the human multidrug transporter proteins, MDR1 and MRP1. Biochim Biophys Acta.

[17] Erlichman C, Boerner SA, Hallgren CG, Spieker R, Wang XY, James CD, Scheffer GL, Maliepaard M, Ross DD, Bible KC, Kaufmann SH (2001). The HER tyrosine kinase inhibitor CI1033 enhances cytotoxicity of 7-ethyl-10-hydroxycamptothecin and topotecan by inhibiting breast cancer resistance protein-mediated drug efflux. Cancer Res.

[18] Wang XK, To KK, Huang LY, Xu JH, Yang K, Wang F, Huang ZC, Ye S, Fu LW (2014). Afatinib circumvents multidrug resistance via dually inhibiting ATP binding cassette subfamily G member 2 *in vitro* and *in vivo*. Oncotarget.

[19] Kitazaki T, Oka M, Nakamura Y, Tsurutani J, Doi S, Yasunaga M, Takemura M, Yabuuchi H, Soda H, Kohno S (2005). Gefitinib, an EGFR tyrosine kinase inhibitor, directly inhibits the function of P-glycoprotein in multidrug resistant cancer cells. Lung Cancer.

[20] Shi Z, Peng XX, Kim IW, Shukla S, Si QS, Robey RW, Bates SE, Shen T, Ashby CJ, Fu LW, Ambudkar SV, Chen ZS (2007). Erlotinib (Tarceva, OSI-774) antagonizes ATP-binding cassette subfamily B member 1 and ATP-binding cassette subfamily G member 2-mediated drug resistance. Cancer Res.

[21] Zheng LS, Wang F, Li YH, Zhang X, Chen LM, Liang YJ, Dai CL, Yan YY, Tao LY, Mi YJ, Yang AK, To KK, Fu LW (2009). Vandetanib (Zactima, ZD6474) antagonizes ABCC1- and ABCG2-mediated multidrug resistance by inhibition of their transport function. PLoS One.

[22] Mi YJ, Liang YJ, Huang HB, Zhao HY, Wu CP, Wang F, Tao LY, Zhang CZ, Dai CL, Tiwari AK, Ma XX, To KK, Ambudkar SV, Chen ZS, Fu LW (2010). Apatinib (YN968D1) reverses multidrug resistance by inhibiting the efflux function of multiple ATP-binding cassette transporters. Cancer Res.

[23] Dai CL, Tiwari AK, Wu CP, Su XD, Wang SR, Liu DG, Ashby CJ, Huang Y, Robey RW, Liang YJ, Chen LM, Shi CJ, Ambudkar SV, Chen ZS, Fu LW (2008). Lapatinib (Tykerb, GW572016) reverses multidrug resistance in cancer cells by inhibiting the activity of ATP-binding cassette subfamily B member 1 and G member 2. Cancer Res.

[24] Tiwari AK, Sodani K, Wang SR, Kuang YH, Ashby CJ, Chen X, Chen ZS (2009). Nilotinib (AMN107, Tasigna) reverses multidrug resistance by inhibiting the activity of the ABCB1/Pgp and ABCG2/BCRP/MXR transporters. Biochem Pharamcol.

[25] Shaw AT, Kim DW, Mehra R, Tan DS, Felip E, Chow LQ, Camidge DR, Vansteenkiste J, Sharma S, De Pas T, Riely GJ, Solomon BJ, Wolf J, Thomas M, Schuler M, Liu G (2014). Ceritinib in ALK-rearranged non-small-cell lung cancer. N Engl J Med.

[26] Locher KP, Borths E (2004). ABC transporter architecture and mechanism: implications from the crystal structures of BtuCD and BtuF. FEBS Lett.

[27] Dai CL, Tiwari AK, Wu CP, Su XD, Wang SR, Liu DG, Ashby CJ, Huang Y, Robey RW, Liang YJ, Chen LM, Shi CJ, Ambudkar SV, Chen ZS, Fu LW (2008). Lapatinib (Tykerb, GW572016) reverses multidrug resistance in cancer cells by inhibiting the activity of ATP-binding cassette subfamily B member 1 and G member 2. Cancer Res.

[28] Dai CL, Liang YJ, Wang YS, Tiwari AK, Yan YY, Wang F, Chen ZS, Tong XZ, Fu LW (2009). Sensitization of ABCG2-overexpressing cells to conventional chemotherapeutic agent by sunitinib was associated with inhibiting the function of ABCG2. Cancer Lett.

[29] Mi YJ, Liang YJ, Huang HB, Zhao HY, Wu CP, Wang F, Tao LY, Zhang CZ, Dai CL, Tiwari AK, Ma XX, To KK, Ambudkar SV, Chen ZS, Fu LW (2010). Apatinib (YN968D1) reverses multidrug resistance by inhibiting the efflux function of multiple ATP-binding cassette transporters. Cancer Res.

[30] Zhou WJ, Zhang X, Cheng C, Wang F, Wang XK, Liang YJ, To KK, Zhou W, Huang HB, Fu LW (2012). Crizotinib (PF-02341066) reverses multidrug resistance in cancer cells by inhibiting the function of P-glycoprotein. Br J Pharmacol.

[31] West KA, Castillo SS, Dennis PA (2002). Activation of the PI3K/Akt pathway and chemotherapeutic resistance. Drug Resist Updat.

[32] Knuefermann C, Lu Y, Liu B, Jin W, Liang K, Wu L, Schmidt M, Mills GB, Mendelsohn J, Fan Z (2003). HER2/PI-3K/Akt activation leads to a multidrug resistance in human breast adenocarcinoma cells. Oncogene.

[33] Zhang JY, Wu HY, Xia XK, Liang YJ, Yan YY, She ZG, Lin YC, Fu LW (2007). Anthracenedione derivative 1403P-3 induces apoptosis in KB and KBv200 cells via reactive oxygen species-independent mitochondrial pathway and death receptor pathway. Cancer Biol Ther.

[34] Fu L, Liang Y, Deng L, Ding Y, Chen L, Ye Y, Yang X, Pan Q (2004). Characterization of tetrandrine, a potent inhibitor of P-glycoprotein-mediated multidrug resistance. Cancer Chemother Pharmacol.

[35] Robey RW, Honjo Y, Morisaki K, Nadjem TA, Runge S, Risbood M, Poruchynsky MS, Bates SE (2003). Mutations at amino-acid 482 in the ABCG2 gene affect substrate and antagonist specificity. Br J Cancer.

[36] Robey RW, Shukla S, Finley EM, Oldham RK, Barnett D, Ambudkar SV, Fojo T, Bates SE (2008). Inhibition of P-glycoprotein (ABCB1)- and multidrug resistance-associated protein 1 (ABCC1)-mediated transport by the orally administered inhibitor, CBT-1((R)). Biochem Pharmacol.

[37] Chen LM, Wu XP, Ruan JW, Liang YJ, Ding Y, Shi Z, Wang XW, Gu LQ, Fu LW (2004). Screening novel, potent multidrug-resistant modulators from imidazole derivatives. Oncol Res.

[38] Shi Z, Liang YJ, Chen ZS, Wang XW, Wang XH, Ding Y, Chen LM, Yang XP, Fu LW (2006). Reversal of MDR1/P-glycoprotein-mediated multidrug resistance by vector-based RNA interference *in vitro* and *in vivo*. Cancer Biol Ther.

[39] Chen LM, Liang YJ, Ruan JW, Ding Y, Wang XW, Shi Z, Gu LQ, Yang XP, Fu LW (2004). Reversal of P-gp mediated multidrug resistance *in-vitro* and *in-vivo* by FG020318. J Pharm Pharmacol.

[40] Fu L, Liang Y, Deng L, Ding Y, Chen L, Ye Y, Yang X, Pan Q (2004). Characterization of tetrandrine, a potent inhibitor of P-glycoprotein-mediated multidrug resistance. Cancer Chemother Pharmacol.

[41] Mi Y, Lou L (2007). ZD6474 reverses multidrug resistance by directly inhibiting the function of P-glycoprotein. Br J Cancer.

[42] Rabindran SK, Ross DD, Doyle LA, Yang W, Greenberger LM (2000). Fumitremorgin C reverses multidrug resistance in cells transfected with the breast cancer resistance protein. Cancer Res.

[43] Ambudkar SV (1998). Drug-stimulatable ATPase activity in crude membranes of human MDR1-transfected mammalian cells. Methods Enzymol.

